# The Cultivation Modality and Barrier Maturity Modulate the Toxicity of Industrial Zinc Oxide and Titanium Dioxide Nanoparticles on Nasal, Buccal, Bronchial, and Alveolar Mucosa Cell-Derived Barrier Models

**DOI:** 10.3390/ijms24065634

**Published:** 2023-03-15

**Authors:** Helene Stuetz, Eva I. Reihs, Winfried Neuhaus, Maren Pflüger, Harald Hundsberger, Peter Ertl, Christian Resch, Gerald Bauer, Günter Povoden, Mario Rothbauer

**Affiliations:** 1 Faculty of Technical Chemistry, Institute of Applied Synthetic Chemistry and Institute of Chemical Technologies and Analytics, Vienna University of Technology, Getreidemarkt 9/163-164, 1060 Vienna, Austria; 2 Karl Chiari Lab for Orthopaedic Biology, Department of Orthopedics and Trauma Surgery, Medical University of Vienna, Währinger Gürtel 18-22, 1090 Vienna, Austria; 3 Competence Unit Molecular Diagnostics, Center Health and Bioresources, AIT Austrian Institute of Technology GmbH, Giefinggasse 4, 1210 Vienna, Austria; 4 Department of Medicine, Faculty of Medicine and Dentistry, Danube Private University, 3500 Krems an der Donau, Austria; 5 Medical and Pharmaceutical Biotechnology, IMC University of Applied Sciences, Am Campus Krems, Trakt G, 3500 Krems an der Donau, Austria; 6 Science, Research, and Development Division, Austrian Federal Ministry of Defence, 1090 Vienna, Austria; 7 CBRN-Defence-Centre, Austrian Armed Forces, 2100 Korneuburg, Austria

**Keywords:** human mucosa models, nanotoxicology, titanium dioxide, zinc oxide, barrier health, barrier integrity

## Abstract

As common industrial by-products, airborne engineered nanomaterials are considered important environmental toxins to monitor due to their potential health risks to humans and animals. The main uptake routes of airborne nanoparticles are nasal and/or oral inhalation, which are known to enable the transfer of nanomaterials into the bloodstream resulting in the rapid distribution throughout the human body. Consequently, mucosal barriers present in the nose, buccal, and lung have been identified and intensively studied as the key tissue barrier to nanoparticle translocation. Despite decades of research, surprisingly little is known about the differences among various mucosa tissue types to tolerate nanoparticle exposures. One limitation in comparing nanotoxicological data sets can be linked to a lack of harmonization and standardization of cell-based assays, where (a) different cultivation conditions such as an air-liquid interface or submerged cultures, (b) varying barrier maturity, and (c) diverse media substitutes have been used. The current comparative nanotoxicological study, therefore, aims at analyzing the toxic effects of nanomaterials on four human mucosa barrier models including nasal (RPMI2650), buccal (TR146), alveolar (A549), and bronchial (Calu-3) mucosal cell lines to better understand the modulating effects of tissue maturity, cultivation conditions, and tissue type using standard transwell cultivations at liquid-liquid and air-liquid interfaces. Overall, cell size, confluency, tight junction localization, and cell viability as well as barrier formation using 50% and 100% confluency was monitored using trans-epithelial-electrical resistance (TEER) measurements and resazurin-based Presto Blue assays of immature (e.g., 5 days) and mature (e.g., 22 days) cultures in the presence and absence of corticosteroids such as hydrocortisone. Results of our study show that cellular viability in response to increasing nanoparticle exposure scenarios is highly compound and cell-type specific (TR146 6 ± 0.7% at 2 mM ZnO (ZnO) vs. ~90% at 2 mM TiO_2_ (TiO_2_) for 24 h; Calu3 93.9 ± 4.21% at 2 mM ZnO vs. ~100% at 2 mM TiO_2_). Nanoparticle-induced cytotoxic effects under air-liquid cultivation conditions declined in RPMI2650, A549, TR146, and Calu-3 cells (~0.7 to ~0.2-fold), with increasing 50 to 100% barrier maturity under the influence of ZnO (2 mM). Cell viability in early and late mucosa barriers where hardly influenced by TiO_2_ as well as most cell types did not fall below 77% viability when added to Individual ALI cultures. Fully maturated bronchial mucosal cell barrier models cultivated under ALI conditions showed less tolerance to acute ZnO nanoparticle exposures (~50% remaining viability at 2 mM ZnO for 24 h) than the similarly treated but more robust nasal (~74%), buccal (~73%), and alveolar (~82%) cell-based models.

## 1. Introduction

At present, the beneficial use of nanomaterials overrates the knowledge and awareness of offsite occurring nanoparticles. Particle penetration in our biological environment and its significance as a potential threat requires a detailed risk assessment in general [1].

Several important global economic activities are attributed as sources of airborne nanoparticles (NPs) ranging from 1 to 100 nm in size including transportation pipelines, agricultural processes, fabric- and clothing manufacturing, metal- and construction industry as well as food and health technologies. Airborne nanoparticles can either be generated intentionally as coatings for electronic component production, in medical, food, and cosmetic applications or unintentionally as aerosol emissions during subtractive manufacturing processes and dissipation events. In the framework of extensive risk analysis and novel detection modalities, also other potential release scenarios are gaining more and more awareness [2,3]. Coating abrasion [4], liposome production [5], as well as braking emissions [6,7], have been identified as heavy nanoparticle sources. Aside from ingestion as well as dermal exposure, inhalation of submicron scale metal entities from a pure character and their compounds as well as non-metal NPs, represent a constantly rising and serious health issue connected with the respiratory system. For any potentially toxic airborne nanomaterial, nasal and/or oral inhalation is the main route of entry [8]. After passing the nasal and oral cavities, aerosols travel through the pharynx, larynx, and trachea reaching bronchi, bronchioles, and finally alveoli. The epithelial tissues that line the respiratory tract include several different cell types that form specialized structures serving essential physiological functions such as blocking unwanted microorganisms and small particles from tissue and bloodstream penetration [9]. While nasal hair restrains particles (≥1 µm) from inhaled air, smaller particles can follow the respiratory system unrestrained where they can interact with epithelial tissue surfaces that are unprotected by physical structures that promote air clearance and hinder particle entry. It is important to note that when NPs leave their route of purpose towards an uncontrolled environment resulting in their subsequent inhalation by living biological systems is generally considered dangerous and labeled as a potential health hazard [10]. For instance, even advanced medical nanomaterials such as anti-bacterial coatings and nano drug delivery systems in anti-cancer therapies or thermotherapies can exhibit significant toxic potentials following an unwanted intake and even from therapy-induced intake. Here, dose- and site-of-entry dependent toxicity is strongly influenced by the intrinsic physicochemical properties of the nanomaterial such as material type, size, surface chemistry, and chemical inertness [11]. Cerium oxide, a prominent industrial process catalyst, or other industrial by-products such as ultrafine carbon can result in a reduction in essential cellular and tissue physiological processes in the lung that can lead to strong inflammatory reactions [12,13]. Additionally, unintended inhalation of zinc oxide and titanium oxide, which are widely applied NPs for ultraviolet (UV) protection in sunscreens, cosmetic powders, electronics, and paints are known to provoke dermal toxicity, dysregulation of immune cells, and potential toxicological effects in lung tissue due to airborne consumption [14,15]. Particle exposure through dermal penetration and ingestion is also an urgent matter of investigation to fully elucidate the yet unknown complete toxicological profile of nano-sized TiO_2_ and ZnO [16]. Aside from the well-known inflammatory properties of titanium nanomaterials, yet elusive aspects of the phototoxic, irritating, and corrosive effects of TiO_2_ on human skin in vitro have also been investigated [17,18,19]. However, murine studies investigating medical TiO_2_ or deriving from the industry point out a strong tendency for it to accumulate in most major organs including the liver, kidneys, and brain [20]. By interfering with hepatocyte mitochondrial functions, cellular apoptosis, and extensive reactive oxygen species (ROS) release in mature tissue, nanoscale TiO_2_ can cause severe developmental issues. Zinc oxide nanomaterials, on the other hand, show a more acute cytotoxic potential and thus higher risk capacity as demonstrated for in vitro studies on the lungs, nervous and digestive systems [21,22,23]. This higher cytotoxic potential can be attributed not only to cytotoxic mechanisms involving such metallic nanoparticles alone but also ion-shedding and the consequential accumulation of toxic free intracellular zinc ions triggering unfavorable events including genetic integrity [24], mitochondrial functionality, [25] as well as cell survival and apoptosis mechanisms including nuclear factor ‘kappa-light-chain-enhancer’ of activated B-cell (NFκB) [26,27] and p53/p38 [22,23,28]).

Inorganic metal nanoparticles including gold (Au), Ag (silver), and Al (aluminum) been applied to push the frontiers of biomedical science and engineering [25,29,30,31]. Au particles are a promising vehicle as a therapeutic tool for diagnostics and drug delivery. Ag at the nanoscale level is another promising bioactive agent with wanted toxic effects due to its physicochemical properties (shapes, sizes, and surface charge), [32] as nanosilver is a potent antibacterial agent that shows severe side effects on human health [33], nonetheless, changes in solubility can be beneficial for targeted delivery as cancer therapy [34]. Al NPs can act neurodegenerative, [35] while its oxide compound intracellularly up taken as Al_2_O_3_ [36] can interfere with intracellular proteins, genomic deoxyribonucleic acid (DNA), and messenger ribonucleic acids (mRNAs) [37]. In general, the accumulation of NPs in organs over time can additionally give rise to a systemic, inflammatory response and interfere with the immune system as shown in animal model studies on Au, Ag as well as Al NPs [38,39,40].

Introduced nanoparticles can further become compromising in terms of overall organ health and functionality by interrupting the barrier integrity of dedicated, protective tissue formations due to the described cytotoxic effects. However, studies that investigate the effect of mentioned NPs are mainly focused on the dermal or oral entry route while inhalation studies are still rare. Moreover, in vitro studies that elaborate on the effect of nanoparticle models on the upper and lower respiratory tract are often limited to submerged culture conditions. In terms of increasing relevance and as an alternative to toxicological animal models, in vitro lung barrier systems exhibiting in vivo-like properties are often used to assess the health effects of airborne nanomaterials [41,42]. Cellular models for the study of NP exposure are essential for regulatory cytotoxic testing, to identify potential hazardous properties and side effects very quickly. Due to their increased robustness, availability, and assay reproducibility, lung epithelial cell lines are generally used in nanotoxicological studies of the respiratory tract instead of primary cells that exhibit only a limited lifespan. Nevertheless, optimization of cell culture conditions that promote cell viability, proliferation, and differentiation towards the formation of a tight barrier is key for conducting nanotoxicological screening including the application of an ALI [43]. Monolayer cultivation does not represent the ideal set-up for the cultivation and maturation of barrier structures from respiratory cells for further testing. Consequently, an ALI setup is important since it closely reflects in vivo conditions of nutrition (basal-barrier-sided physiological medium supply) and inhalation (particle exposure at the apical pole of the associated cell layer) in the lower respiratory tract where epithelial surfaces are covered by a thin liquid layer keeping the cells in a moist microenvironment [44]. This means that only an ALI system can properly simulate physiological-relevant toxicant or particle exposures [42]. A comparative study investigating submerged lung cell cultures in a LLI, with ALI culture of lung cells, and rat models demonstrated that ALI most closely represented the in vivo results following exposure to toxicants as indicated by similar cell viability and release of lung inflammation markers [43]. Additionally, ALI proved to be a very useful tool in toxicity and pharmacological studies of inhaled particles, revealing significant differences in the secretion of pro-inflammatory interleukin-8 (IL-8) and oxidative stress to LLI upon ZnO nanoparticle exposure [44]. The sensitivity of an ALI was also tested as superior in contrast to submerged cultures [45]. Apart from the further refinement of the cellular components, the development of new cell lines [46] and the optimization of exposure systems for predictive studies are essential to improve result validity. For mimicking in vivo conditions, particle exposure in form of aerosol droplets combined with an ALI setup increased the test sensitivity for an inhalation toxicity model [47]. Furthermore, a platform capable of mimicking breathing motion including associated air flow and heating of the sample gas during exposure to adherent lung epithelial cultures was reported in an attempt to best emulate the biotransformation of the exposed substances [48]. Another approach towards a robust and reliable test platform that uses the cyclic in vitro cell-stretch bioreactor for the conditioning of alveolar A549 cells for a breathing-like motion during barrier maturation [49]. It is, however, important to note that in addition to optimum physiologically-relevant culture conditions the choice of cell lines and applied bioactive compounds such as hormones are also critical in promoting reliable barrier formation. Potential standardization of ALI-supported models in terms of cultivation conditions is likewise a matter of interest [50] to increase the inter-lab model and result in reproducibility while decreasing result variability using the same modeling approach.

To gain a deeper understanding of the complex interplay between cell culture conditions, media composition, and cell types in nanotoxicological studies, four human tissue barrier models using nasal (RPMI2650), buccal (TR146), alveolar (A549), and bronchial (Calu-3) mucosal cell lines are investigated using transwell-based cultivation at the liquid-liquid as well as air-liquid interface. Considering the entry route of airborne toxicants and their location as well as particle-exposed respiratory epithelial tissues, a higher degree of in vivo-like properties can be reached by including differential epithelial cells lines deriving from first-contact tissues of the upper [51,52] (nasal and buccal) and lower [53,54] (bronchial and alveolar) respiratory tract. As the current study is part of a bigger project framework to develop a multi-mucosa on-a-chip system for nanotoxicological studies, the initial characterizations here were conducted with a shared medium formulation. Initially, cell size, confluency, tight junction localization, as well as barrier formation is monitored using TEER-measurements throughout an incubation period of 22 days in the presence and absence of corticosteroids (e.g., dexamethasone or hydrocortisone). Furthermore, the contribution of initial barrier confluency on nanomaterial dose-response behavior is investigated by analyzing the effect on cellular viability after 24 h of nanomaterial exposure to initial 50% and 100% confluent barriers in submerged cultures using the Presto Blue assay. Finally, the effect of nanomaterial exposure is investigated using immature liquid-liquid mucosa barriers at day 5 and compared to the response of the maturated mucosa barriers at day 22 of cultivation under air-liquid interface conditions using TEER and Presto Blue assays. One intention of the current comparative study is the detailed characterization of the main mitigating factors known to influence nanotoxicological screening efforts, thus fostering better guidelines for improved harmonization and standardization of advanced in vitro cell-based assays for inhalation studies.

## 2. Results

### 2.1. Initial Characterization and Seeding Density Optimization of the Four Mucosa Cell Lines

**Figure 1:**
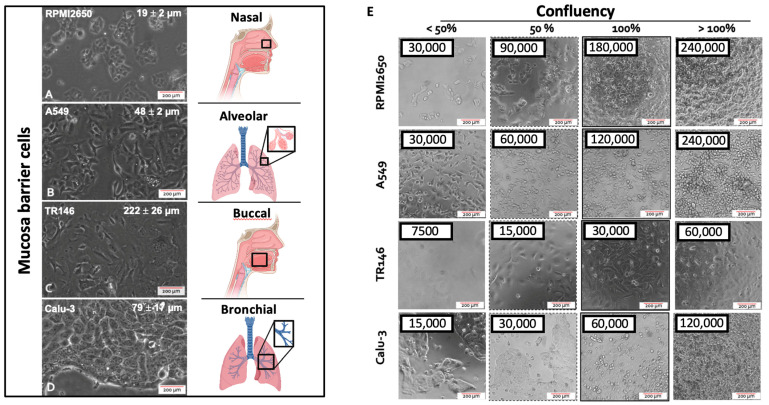
(**A**–**D**) Representative phase contrast images of cell morphology of the four different mucosa cell lines including (**A**) RPMI2650 (nasal), (**B**) A549 (alveolar), (**C**) TR146 (buccal), and (**D**) Calu-3 (bronchial) grown as conventional 2D monocultures in 75 cm^2^ cell culture flasks. (**E**) Representative phase contrast images of the four epithelial mucosal cell lines at various monolayer confluency resulting from variations of initial cell seeding densities 24 h post-seeding in cell culture-treated 96-well plate format with a total growth area of 0.33 cm^2^.

The first parameter to be investigated in any cell-based assays is concerned with optimizing the seeding density needed to reliably establish 100% confluency for each cell type that leads to adequate barrier functionality and health. This aspect is of particular importance since inadequate seeding densities result in extended cultivation periods required to form healthy human barrier models featuring high barrier health and integrity. To study the growth characteristics of employed four mucosal epithelial cell lines, cell size, and confluency, as well as the doubling time of the four epithelial cell lines is initially investigated. The average cellular length was quantified using ImageJ microscopic image analysis, while the confluency was determined experimentally via cell counting and conducting cell titration experiments. As shown in Figure 1, microscopic evaluations highlight differences in cell sizes in the range of approximately 20 to over 200 µm exhibiting a characteristic polygonal epithelial cell morphology. As these significant differences in cell size between the individual tissue types impact the initial cell seeding density needed to establish dense two-dimensional cell layers, the next set of experiments analyzed the effect of increasing initial cell seeding densities on barrier confluency.

As shown in Figure 1A, RPMI2650 epithelial cells derived from squamous cell carcinoma of the nasal mucosa showed a tendency to grow in small patches. The very small ovoid to squamous appearing cells displayed an average size of 19.3 ± 2.3 µm reaching confluency of approximately 95% at an initial cell seeding density of 6 × 10^5^ cells/cm^2^. In turn, A549 cells, which are epithelial cells of adenocarcinoma of the alveolar lung epithelium, revealed a characteristic spindle-like morphology when seeded below confluency with an average size of 47.4 ± 5.2 µm. Growing in bigger patches this alveolar cell type reached confluency at an initial seeding density of 4 × 10^5^ cells/cm^2^. Interestingly, TR146, a squamous cell carcinoma cell line of the buccal epithelium displayed the biggest cell size of 222.3 ± 25.9 µm producing cell monolayers when seeded at approximately 1 × 10^5^ cells/cm^2^; while Calu-3 being a bronchial adenocarcinoma cell line displayed an average size of 79.1 ± 16.6 µm with confluency at a seeding density of 1.8 × 10^5^ cell/cm^2^. Summarizing the performance indicators in Table 1, the optimized initial cell seeding densities were used in all subsequent experiments in microtiter plates as well as transwell insert approaches to provide each cell type the best starting conditions to form tight human cell barriers taking significant cell-specific variations in both cell size as well as growth capacities into account.

### 2.2. Optimization of the Liquid-Liquid Interface (LLI)-Based Cultivation Approach and Transfer to Air-Liquid Interface (ALI) Cultivation of Human Mucosa Barrier Models

**Figure 2:**
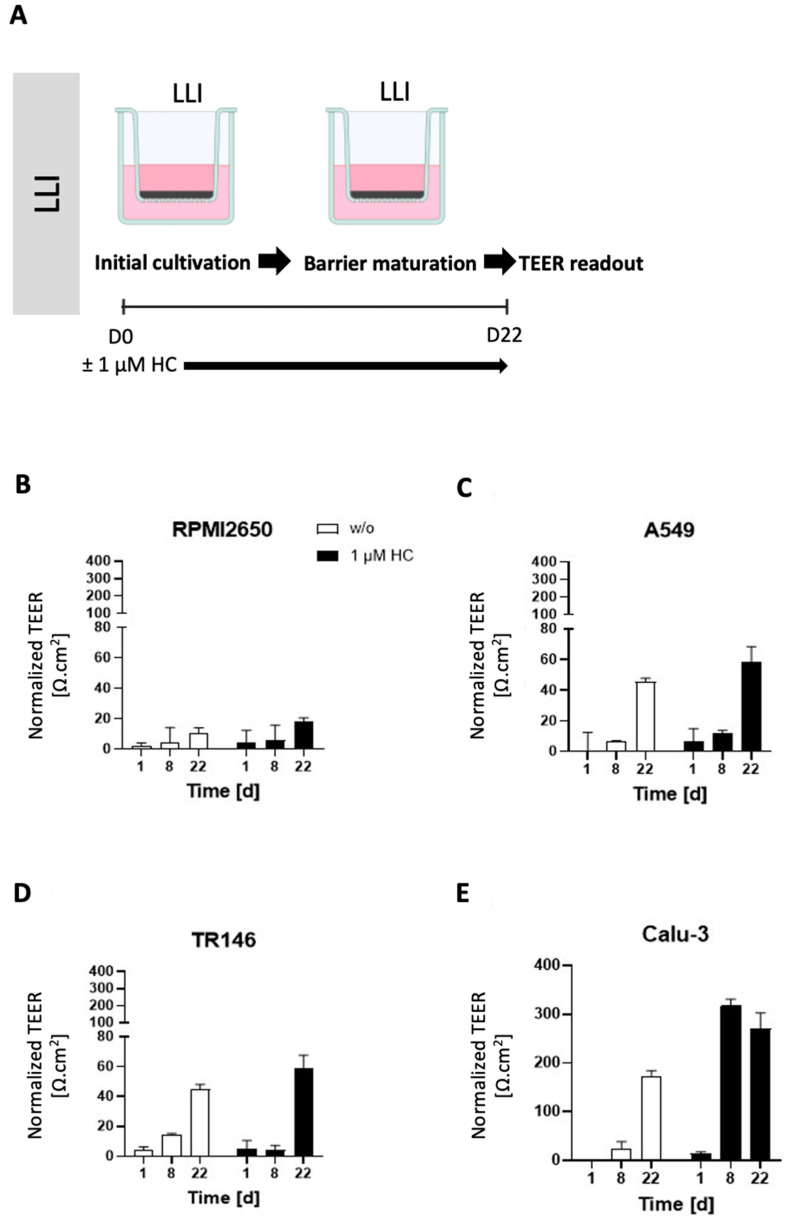
Effect of hydrocortisone (HC) on TEER-based read-out of barrier maturation dynamics of mucosa cells grown at confluency on porous membrane inserts throughout 22 days of liquid-liquid interface (LLI) culture. (**A**) Graphical visualization of the cultivation and treatment protocol, and normalized time-resolved TEER analysis of (**B**) RPMI2650, (**C**) A549, (**D**) TR146, and (**E**) Calu-3. Data are expressed as mean ± standard deviation for *n* = 3 replicates.

As a next step, the initially optimized protocols for the establishment of tight monolayers of the four individual cell lines were transferred and reevaluated for LLI as well as ALI interface cultivation approaches. Both methods benefit from using a porous membrane as a cultivation surface for epithelial barrier cell growth, where the separation into apical and basolateral cell culture compartments allows for cell polarization that more closely mimics the physiological properties. The additional introduction of an air-liquid interface further induces cell differentiation and barrier maturation. To monitor barrier formation in more detail, in the next set of experiments TEER-monitoring was performed as a non-invasive in situ approach to evaluate barrier integrity as it efficiently detects tight junction formation dynamics in the presence and absence of corticosteroid additives as well as ALI. First, barrier maturation was investigated at the LLI, where the medium is supplied in the basal as well as apical compartments. To increase tight junction formation and thus barrier maturation capacity, hydrocortisone was supplied to the complete medium at an effective concentration of 1 µM with TEER recordings being performed over a total cultivation duration of 22 days on collagen I-coated Transwell inserts. Data from Figure 2 confirm that barrier maturity and integrity improved over time for both treated and untreated barrier models of any cell type (*p* < 0.05; two-way ANOVA with mixed effects analysis test). During the first 8 days of cultivation, corticosteroid treatment only showed slightly increased barrier maturation capacities (*p* > 0.05), with overall barrier formation boosted only for Calu-3 barrier models when treated with hydrocortisone compared to the respective untreated controls (*p* < 0.005). The least improvement in barrier maturation by supplementation with hydrocortisone was observable for RPMI2650 (Figure 2A), followed by A549 and TR146 which all together showed no TEER improvement (*p* > 0.05). Notably, the bronchial barrier model established with Calu-3 cells was affected by hydrocortisone treatment and improved overall TEER values after 8 days of cultivation at the LLI resulting in an overall increase of TEER from 173.5 ± 11.1 Ω·cm^2^ to 270.4 ± 32.4 Ω·cm^2^ within 8 days (*p* < 0.005). Even though in earlier time points the barrier maturation was faster for the hydrocortisone-treated Calu-3 barriers, both treated and untreated Calu-3 cells reached comparable final TEER values after 22 days in LLI conditions.

**Figure 3:**
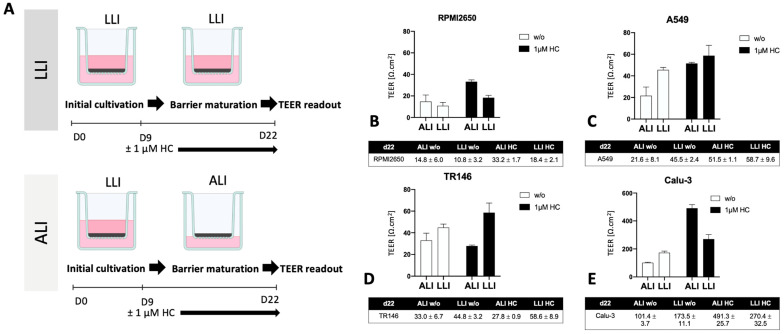
Impact of ALI compared to LLI cultivation on TEER in the presence and absence of hydrocortisone. (**A**) Visualized protocol for the LLI and ALI cultivation of mucosa cells grown on porous membrane inserts over a period of 22 days. (**A**–**C**) Comparative analysis of LLI with ALI cultivation (initial LLI cultivation period of 8 days followed by 14 days of barrier maturation at the ALI) in the presence and absence of hydrocortisone (HC) for (**B**) RPMI2650, (**C**) A549, (**D**) TR146, and (**E**) Calu-3. Data are expressed as mean ± standard deviation for *n* = 3 replicates.

Next, the impact of ALI conditions on the maturation capacities of human barrier model was investigated using a similar setup as the above corticosteroid experiments. To determine whether an ALI boosts barrier functionality, the apical medium was removed after 8 days of submerged cultivation, to allow the cell layers to continue to be grown at ALI conditions with basolateral medium exchange every other day for another 14 days. As a side note, for the successful establishment of ALI conditions, some optimizations in the total volume of the basal medium compartment were necessary by switching from conventional Costar™ microtiter plates to more specialized thincert^®^ plates, where 4 mL of the basal medium could be supplied at ALI conditions to provide a comparable amount of total medium to provide similar nutrient supply frequencies of every other day with regards to the optimized LLI cultivation protocols used in prior Transwell experiments. As indicated in Figure 3A, TEER values of nasal RPMI2650 barrier models improved from 10.8 ± 3.2 Ω·cm^2^ at LLI to ALI: 14.8 ± 6.0 Ω·cm^2^ for ALI-based barrier maturation protocols with further improvement for the 1 µM hydrocortisone treatment boosting TEER values up to 33.2 ± 1.7 Ω·cm^2^ at ALI at day 22 post-seeding. In contrast, for the alveolar A549 barrier model shown in Figure 3B, the LLI tended to generally show higher TEER values (LLI: 45.5 ± 2.4 Ω·cm^2^, and ALI: 21.6 ± 8.1 Ω·cm^2^). Hydrocortisone treatment was effectively boosting TEER for both cultivation protocols and approximated both techniques to 58.7 ± 9.6 Ω·cm^2^ at LLI and 51.5 ± 1.1 Ω·cm^2^ at ALI. Similarly, TR146 also showed increased barrier integrity when cultured at LLI as shown in Figure 3C (*p* < 0.05; two-way ANOVA with multiple comparisons test) yielding TEER values of 44.8 ± 3.2 Ω·cm^2^ for LLI compared to 33.0 ± 6.7 Ω·cm^2^ for ALI conditions. Additionally, TR146 barriers were unaffected by hydrocortisone for the ALI culture technique (27.8 ± 0.9 Ω·cm^2^). Again, alveolar Calu-3 barriers showed by far the highest barrier integrity, with 173.5 ± 11.1 Ω·cm^2^ for LLI and 101.4 ± 3.7 Ω·cm^2^ for ALI as shown in Figure 3D (*p* < 0.0001). Moreover, hydrocortisone treatment boosted barrier maturation significantly up to final TEER values of 270.4 ± 32.5 Ω·cm^2^ and 491.3 ± 25.7 Ω·cm^2^ for LLI and ALI, respectively. For the sake of comparability in all subsequent experiments, 1 µM hydrocortisone (HC) was added to all mucosa cell models.

### 2.3. Impact of Barrier Cell Confluency on the Dose-Response Readout of Human Barrier Models Exposed to TiO_2_ and ZnO Nanoparticles

**Figure 4:**
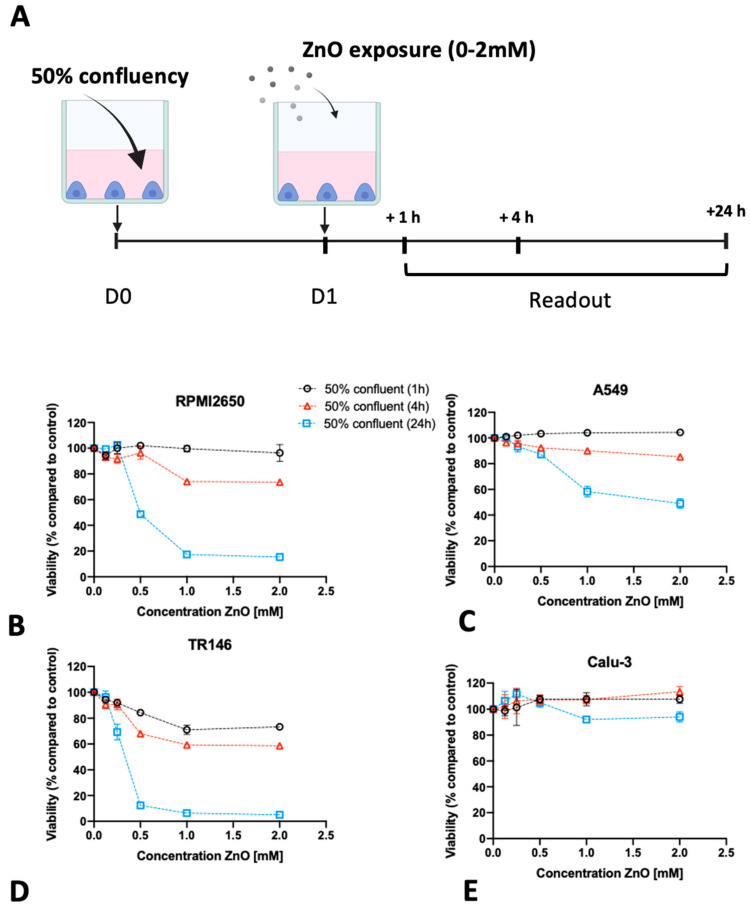
(**A**) Visual representation of the assay protocol for investigating the impact of 50% initial seeding density on nanotoxicological readout using Presto Blue viability assay. (**B**–**E**) ZnO nanoparticle toxicity of immature barrier cell monolayers in conventional cell culture-treated microtiter plates at 50% cell confluency, 24 h post-seeding, using a Presto Blue assay at 1, 4, and 24 h post-exposure to 70–90 nm sized, uncoated ZnO NPs in for (**B**) RPMI2650, (**C**) A549, (**D**) TR146, and (**E**) Calu-3. Data are expressed as mean ± standard deviation for *n* = 3 replicates.

Following the establishment of optimum seeding density on barrier maturation for each of the four mucosal cell lines in the first section, the following investigations focused on the dose-time responses of lung, nasal, and buccal cell barrier models using two well-established nanoparticle types. The four barrier cell models were plated at 50% and 100% confluency and incubated for different exposure times up to 24 h in the presence of increasing concentrations of ZnO and TiO_2_ nanoparticle solutions. Cell viability was determined after 1, 4, and 24 h using the Presto Blue assay. Figure 4 shows the effect of ZnO nanoparticles for each cell line using an immature state at 50% confluency. A general trend was observable where incubation times of 4 h already resulted in compromised cell viability at high concentrations above 1 mM for ZnO nanoparticle exposure. Moreover, longer incubation times of 24 h enhanced this initial cytotoxic response and showed cell viability decreased significantly starting from 0.5 mM ZnO NPs. For RPMI2650, the cell viability declined to 74 ± 1.7% at 1 mM and to 73 ± 1.8% at 2 mM ZnO NPs at 4 h. At 24 h cell viability was compromised to 68 ± 6.2% at concentrations of 0.5 mM, 38 ± 0.4% at 1 mM, and 26 ± 2.6% at 2 mM (Figure 4A). For A549, a slight increase in cell viability was observed at 1 h incubation indicating most probably an initial stress response. After 4 h, the cell viability dropped to 90 ± 1.0% at 1 mM and 85 ± 0.6% at 2 mM, which was further exacerbated within 24 h the cell viability further dropped to 87 ± 2.5% at 0.5 mM, 58 ± 4.2% at 1 mM and 49 ± 3.6% at 2 mM (Figure 4B). For TR146 the incubation period of 1 h already showed a cytotoxic effect of 84 ± 1.4% at 0.5 mM, 71 ± 3.6% at 1 mM, and 73 ± 2.2% at 2 mM, which intensified in longer incubation times. For 4 h cell viabilities of 68 ± 1.3% at 0.5 mM, 59 ± 1.3% at 1 mM, and 59 ± 2.0% at 2 mM were determined. After 24 h cell viability was further decreased to 12 ± 0.7% at 0.5 mM, 5 ± 0.8% at 1 mM, and 6 ± 0.7% at 2 mM (see Figure 4C). Interestingly, the bronchial barrier Calu-3 model showed the lowest susceptibility towards ZnO-based toxic events with a slight increase in cell viability after 1 h for concentrations higher than 0.5 mM (Figure 4D), 107.6 ± 3.3% for 2 mM, 107.9 ± 5.1% for 1 mM, and 107.6 ± 2.6% for 0.5 mM. In contrast to the three other immature barrier models, this trend was continued at 4 h post-incubation (2 mM: 113.6 ± 3.9% and 1 mM: 107.3 ± 3.0% viability). However, after 24 h of ZnO nanoparticle exposure cell viability for concentrations above 1 mM started to decline (2 mM: 93.9 ± 4.21% and 1 mM: 91.96 ± 0.28% viability), while the slight increase in cell viability at lower concentrations again may be attributed to cell stress rather than cytotoxic events.

**Figure 5:**
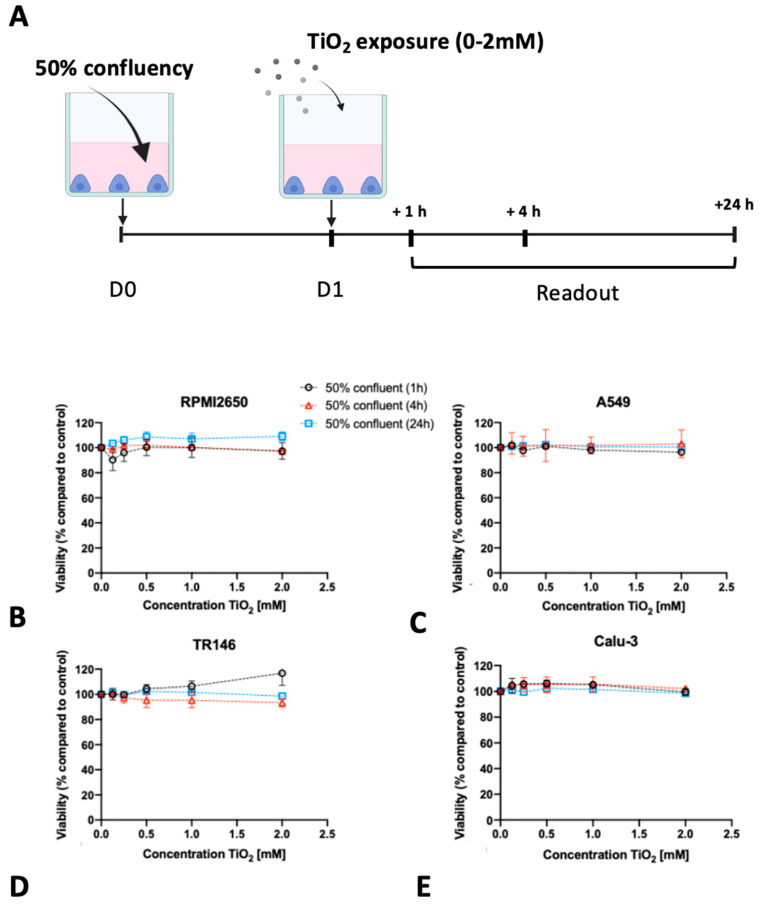
(**A**) Visual representation of the assay protocol for investigating the impact of 50% initial seeding density on nanotoxicological readout using Presto Blue viability assay. (**B**–**E**) TiO_2_ nanoparticle toxicity in submerged cultures at 50% confluency, 24 h post-seeding. Cell viability was measured using a Presto Blue assay at 1, 4, and 24 h post-exposure to 5–6 nm sized, uncoated TiO_2_-NPs, graphical timeline in (**A**). (**B**) RPMI2650, (**C**) A549 (**D**) TR146 and (**E**) Calu-3. Data are expressed as mean ± standard deviation for *n* = 3 replicates.

In contrast to ZnO nanoparticles which were selected for the well-reported cytotoxic effects of both zinc oxide particles as well as secreted zinc ions, TiO_2_ nanoparticles did not negatively affect the viability of the immature cell models (see Figure 5). For RPMI2650, a slight increase in cell viability was observed in the samples treated for 24 h with TiO_2_ nanoparticles. An increase in viability was also observed for TR146, after 1 h treatment, whereas for 2 mM TiO_2_ nanoparticles cell viability of 117 ± 10% was observed. For A549 and Calu-3 neither up-now downward change in cell viability was observed independent of concentrations throughout the entire 24 h incubation period with TiO_2_-NPs.

**Figure 6:**
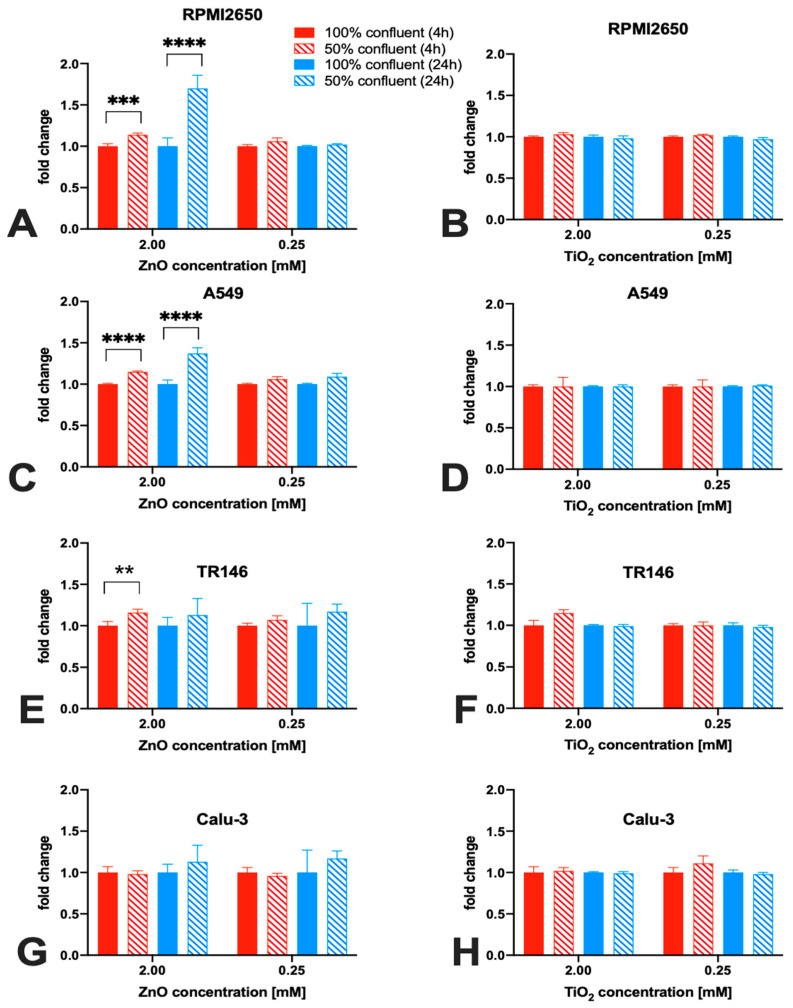
The impact of cell confluency on nanoparticle cytotoxicity of four mucosal cell lines including (**A**,**B**) RPMI2650, (**C**,**D**) A549, (**D**,**E**) TR146, and (**F**,**G**) Calu-3. Cell viability was measured using a Presto Blue assay at 4 and 24 h post-exposure to 80 nm sized, uncoated ZnO NPs (**A**,**C**,**E**,**G**) as well as 5 nm sized, uncoated TiO_2_ NPs (**B**,**D**,**F**,**H**). Data are expressed as mean ± standard deviation for *n* = 3 replicates. Two-way ANOVA corrected with a Tukey test: ** *p* < 0.02, *** *p* < 0.0002, **** *p* < 0.0001.

It has to be noted that most investigations of nanoparticle toxicity in conventional studies have been performed for a variety of cell lines seeded frequently at sub-confluent levels, thus making a comprehensive evaluation based on published data on the effect of barrier maturation stages almost impossible. Consequently, the impact of barrier maturity on cytotoxicity was investigated in more detail in the next set of experiments by exposing mucosal cell lines established at 50% or 100% confluency with both nanoparticle types for up to 24 h. As shown in Figure 6, significant differences in the cytotoxic response upon ZnO NP treatment were observed only with incubation times of 4 and 24 h and in the presence of concentrations of 2 mM ZnO NPs. For RPMI2650 (Figure 6A) this difference was significant for 4 h, with 1.14 ± 0.02-fold higher cytotoxicity in 50% confluency (*p* < 0.0002) and a 1.70 ± 0.16-fold higher cytotoxicity after 24 h (*p* < 0.0001). Furthermore, a similar picture was seen for A549 (Figure 6C), where after 4 h incubation time a 15% increase in cytotoxic effect was observed (*p* < 0.0001), whereas, for the 24 h incubation period, a 1.37 ± 0.07 times higher effect was observed in cultures of 50% confluency (*p* < 0.0001). Contrarily, TR146 shows a different trend (Figure 6E).

A significant difference in cytotoxicity between 50% and 100% coverage of growth area was only observed after 4 h of incubation time, with a change of 16% (*p* < 0.02). For Calu-3 (Figure 6G), no significant difference (*p* > 0.1) in cytotoxicity could be observed in correlation with the confluency (2 mM: 1.02 ± 0.04-fold higher in 50% confluent culture after 4 h and 1.04 ± 0.03 after 24 h). A similar trend was observed for the 0.25 mM concentration, with a 1.11 ± 0.09-fold higher cytotoxicity in 50% confluent culture after 4 h (*p* > 0.31) and was lower after 24 h (0.95 ± 0.01-fold; *p* > 0.1). For TiO_2_ nanoparticles, no significant difference in cytotoxicity for any of the four mucosal cell lines was observed in dependence of confluency (Figure 6B, D, F, H).

### 2.4. Toxicological Evaluation of Mature Mucosa Barrier Models Challenged with Subtoxic and Toxic ZnO and TiO_2_ Nanoparticle Concentrations

**Figure 7:**
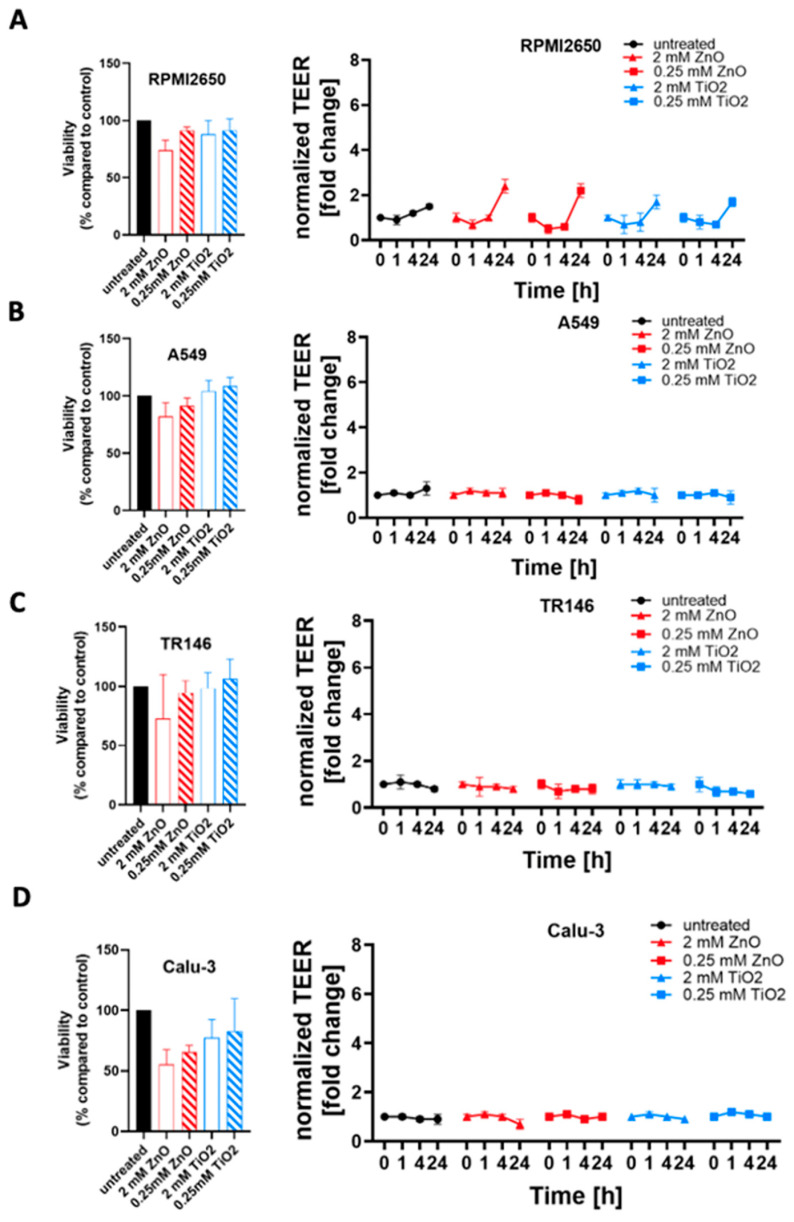
Comparison of zinc oxide and titanium dioxide nanoparticle toxicity in dependence of culture technique. Cell viability was measured after 24 h incubation with nanoparticles, measured using a Presto Blue Assay, (**A**) RPMI2650, (**B**) A549, (**C**) TR146, and (**D**) Calu-3. Data are expressed as mean ± standard deviation for *n* = 3 replicates. Two-way ANOVA with Tukey posthoc multi-comparisons test.

To further investigate the modulating effects of tissue microphysiology on barrier integrity, the four mucosa barrier models cultivated using the optimized ALI protocols were treated with ZnO or TiO_2_ nanoparticle solutions. In a comparative study, mucosa barrier toxicity data were collected using ALI and submerged monolayer cultures throughout 24 h of exposure to ZnO and TiO_2_ nanoparticles. In Figure 7, the viability results of submerged cultures (100% confluency in cell culture-treated microtiter plates) are compiled using viability and TEER readouts. As shown in Figure 7A and Figure S1A, cytotoxicity of 2 mM ZnO NPs was highest in RPMI2650 barriers including submerged cultures (26.0 ± 2.6% viability) and mature barrier models (74.0 ± 8.8% viability). For the lower exposure concentration of 0.25 mM, ZnO NPs showed a stress response in submerged cultures (104.3 ± 0.6% viability) which was not observable for mature barrier models, and higher toxicity in the barrier models (91.5 ± 3.0% viability). TiO_2_ exposure showed similar stress responsiveness to the conventional 2D monolayer model for a 2 mM concentration (submerged: 107.0 ± 1.7% viability vs. mature: 87.9 ± 11.9% viability) as well as the 0.25 mM nanoparticle concentration (submerged: 103.3 ± 0.6% viability vs. mature: 91.4 ± 10.0% viability). Overall, both TiO_2_ and ZnO showed an initial decline in barrier integrity (*p* < 0.005) followed by a strong increase in TEER (*p* < 0.0001) relative to non-exposed control. Figure 7B and Figure S1B indicate, that A549 cultures show the lowest viability in submerged cultures treated with 2 mM ZnO NPs (67.3 ± 3.1% viability compared to the mature barrier (82.1 ± 12.0%). For 0.25 mM ZnO NPs, RPMI2650 cultivated under submerged and ALI conditions showed no toxicities with 102.7 ± 0.6% viability vs. 91.3 ± 6.9% for submerged and ALI samples (*p* > 0.05). A similar trend was observed for both TiO_2_ nanoparticle exposure scenarios: The 2 mM concentration exposure resulted in viability of 100.3 ± 0.6% in submerged cultures and 104.2 ± 9.3% in the mature barrier model. At a 0.25 mM TiO_2_ concentration, viabilities of 102.7 ± 0.6% in submerged cultures versus 108.4 ± 7.7% for the mature barrier model were observable. Figure 7C and Figure S1C further confirm the overall lower cytotoxic response of mature barriers also for the TR146 cell model, where the submerged cells resulted in a viability of 5.7 ± 0.6% after 24 h incubation with 2 mM ZnO while the mature barrier demonstrated a viability decline to 73.1 ±26.8% (*p* < 0.0001). TiO_2_ NP exposure resulted in a slight trend for viability increase for mature barrier models (106.1 ± 14.1% for 2 mM and 106.1 ± 14.1% for 0.25 mM). Even though the viability read-out for 2 mM ZnO nanoparticles indicated cell death, the barrier integrity analyzed by TEER was unaffected (*p* > 0.05). In contrast to the first three mucosa barrier models, Calu-3 cells showed responses similar to the 2D mono cultivation approach analyzed before again a quite different response (see Figure 7D and Figure S1D). As shown in Supplementary Material Figure S1D, cell viability was more affected in the mature barrier model, than in the submerged culture with 95.3 ± 0.6% at 2 mM ZnO for submerged monolayers vs. 55.5 ± 21% for the mature barrier (*p* < 0.05). This was also reflected in a significant decline of TEER values by 30% over 24 h of exposure (*p* < 0.01) found with the ALI conditions. Matured Calu-3 barriers showed no significant decline of cell viability in the range of 77.4 ± 15.1% and 82.8 ± 27.0% when exposed to 2 mM and 0.25 mM TiO_2_ nanoparticles (*p* > 0.1), respectively. Nonetheless, this stress response after 1 h of exposure resulted in a TEER increase of 20% that again leveled off around 90% of the initial resistance values (*p* > 0.05) after 24 h of exposure indicating most probably barrier regeneration processes.

## 3. Discussion and Prospects

The human airway is exposed to thousands of liters of air, and therefore, to a multitude of toxicants daily. Airborne nanomaterials, such as ZnO and TiO_2_ nanoparticles are formed as a side product of many industrial processes and are known to increase over time due to accumulation in the environment [11]. The investigation of nanomaterial-biology interactions and the potentially toxic effects on human barriers are of high importance to understanding their impact on human health. The current work focused on the establishment of four airway mucosal cell lines (nasal, buccal, alveolar, and bronchial epithelium) as mature barrier models at the air-liquid interface for acute toxicological evaluation of nanoparticles under physiologically more relevant culture conditions. ZnO and TiO_2_ nanoparticle toxicity was compared within multiple culture conditions and techniques. Additionally, the beneficial effect of hydrocortisone which has previously been reported to support barrier integrity by aiding the assembly process of zona occludens-1(ZO-1) to other proteins of the junction complex, [55] in turn boosts barrier resistances investigated by TEER. To successfully optimize a set of four mucosa barrier models for nanotoxicological studies, firstly cell characteristics, such as growth conditions, doubling time, size of cells, and corresponding confluency conditions were evaluated and matched to those found in literature as well as general information provided by cell supplies (i.e., the ATCC). The use of RPMI2650 cells as a valid nasal mucosa barrier model was demonstrated previously in other studies, showing excellent application potential, and justifying the choice for this specific cell line [56]. A similar reasoning stands for TR146, a buccal mucosa cell line that was previously optimized to be used as a barrier model at the air-liquid interface by Lin et al., thus presenting a valid barrier model of the oral mucosa [57]. The choice of A549 as a barrier model for toxicity testing may seem unpopular at first, given the many reported limited barrier formation properties of A549 [58]. However, provided the high frequency of applications in toxicological screening, this cell line represents a well-established model for human alveolar epithelium that can be used for cross-study comparisons [13,59,60,61,62]. In turn, the Calu-3 cells are a more obvious choice as a barrier model for the bronchial mucosa due to their excellent barrier maturation and tight junction formation properties [63,64,65,66]. Notably, Calu-3 cells have also been reported previously to form more physiological barrier models of the lung epithelium in comparison to other bronchial epithelial cell lines such as NHBE or NL-20 [67].

Barrier integrity results analysed by TEER in the currentstudy showed that RPMI2650 barriers exhibited TEER values that are close to those of excised nasal mucosa samples [68,69], and are also comparable with TEER values reported in previous studies [70]. In previous studies, TR146 cells formed stronger barriers at LLI compared to ALI [71,72]. Calu-3 further outperformed the other three mucosal cell lines in terms of barrier maturation potential yielding the highest TEER values when cultivated under ALI conditions at comparable orders of magnitude reported previously [63,73]. Barosova et al. already discussed that TEER values at LLI as well as ALI protocols are subject to high inter-laboratory variability, [74] which can be attributed to the observed lower TEER values. Notably, we decided for the current study to use a common media supplement formulation not only for the work conducted in this subproject but also as a collective starting point for potential simultaneous on-chip co-cultivation. Further optimizations of media conditions and supplements, as well as harmonization of individual ALI protocols, are currently under investigation in the next project phase of on-chip integration to optimize the individual barrier properties.

Building from the aspect of barrier formation, the effect of different culture conditions on the toxicity of applied ZnO and TiO_2_ nanoparticles was investigated. Notably, in our study confluency and maturity significantly affected the cytotoxic profile of ZnO nanoparticles in three out of the four mucosal cell lines. As discussed by Heng et al. there are two major factors influencing nanoparticle toxicity in submerged cell culture including firstly, the nanoparticle per cell ratio and secondly the nanoparticle to culture surface area ratio [75]. Even though the fact that individual cells within a confluent monolayer can potentially be less exposed to nanoparticles and that junctions in between cells, secreted cytokines, and growth factors could also dampen the cytotoxic effect, in our current study both nanoparticle diameters below 100 nm were selected to allow active as well as passive nanomaterial uptake, which makes transport inhibition phenomenon over a course of 24 h very unlikely.

Overall, when comparing submerged toxicity data of frequently used sub-confluent cells and the transwell barrier model toxicity responses are challenging because barrier models are more often used for material transport studies than detailed nanotoxicology studies. Even though both constitute viable and well-established models, in our opinion future studies must investigate a variety of human mucosa models in more comprehensive comparative studies, as highlighted by our current study, using more physiological approaches such as ALI protocols. As demonstrated by Leroux et al. air-liquid interface compared to submerged cultures more closely mimics gene expression levels upon TiO_2_ nanoparticle exposure to that in vivo [76,77]. Here, Calu-3 cells showed the least cytotoxic effects upon ZnO exposure, which aligns well with comparable other studies of submerged cultures [78]. Most importantly, the type of barrier cell severely affected the nanotoxicological results in the current study. For instance, when comparing cell-specific results using the optimized ALI culture protocols in the current study Calu-3 bronchial barriers showed less tolerance to zinc oxide nanoparticles compared to established buccal, nasal, and alveolar models. We could highlight the importance of barrier cultivation state when conducting toxicological nanoparticle studies interfacing with human tissue models. Furthermore, conducting such studies under defined and comparable cultivation conditions as demonstrated here, for multiple tissue models allows for potential future harmonization on how to reproducibly perform nanomaterial interaction studies with higher lab-to-lab comparability in tissue-specific analyses.

## 4. Materials and Methods

### 4.1. Cell lines and Culture Conditions

To replicate the pathway of inhaled toxicants, four key cell lines originating from the respiratory system were used:

- RPMI2650, (Sigma-Aldrich, Vienna Austria, Cat. Nr. 88031602) originating from squamous cell carcinoma of nasal epithelium, was cultured in EMEM (Sigma-Aldrich, Vienna, Austria, Cat. Nr. M0325);
- A549 (ATCC, Manassas, Virginia, USA, Cat. Nr. CCL-185) originating from adenocarcinoma from alveolar lung epithelium, were cultured in RPMI1640 (Sigma-Aldrich, Vienna, Austria, Cat. Nr.R8758);
- TR146, (Sigma-Aldrich, Vienna, Austria, Cat. Nr. 10032305) originating from squamous cell carcinoma of buccal (oral) epithelium, were cultured in Ham’s F12 (Sigma-Aldrich, Vienna, Austria, Cat. Nr. 51651C);
- Calu-3, (Sigma-Aldrich, Vienna, Austria, Cat. Nr. HTB-55, kindly provided by IMC FH Krems) originating from adenocarcinoma from bronchial lung epithelium, were cultured in EMEM (Sigma-Aldrich, Vienna, Austria, Cat. Nr. M0325).

All cell culture media were supplemented with 10% fetal bovine serum (FBS; Sigma-Aldrich, Vienna Austria, Cat. Nr. F0804) and 1% Antibiotic- Antimycotic-solution (Sigma-Aldrich, Vienna Austria, Cat. Nr. A5955). Cells were kept in culture, humidified 37 °C, 5% CO_2_, in TC-treated 75 cm^2^ flasks (Greiner Bio-One, Kremsmünster, Austria, Cat. Nr. 658175) and split twice a week by trypsinization (0.5 g/L Trypsin–0.2 g/L EDTA, Sigma-Aldrich, Vienna, Austria, Cat. Nr. T3924). Growth properties and morphology were monitored during the cultivation of cells via light microscopy and counting with an improved Neubauer hemocytometer.

### 4.2. Barrier Formation Studies 

Cells were seeded at 100% confluency, on Thincerts^®^ (Greiner Bio-One, Kremsmünster, Austria, Cat. Nr. 665610, 3 µm pore size and Cat. Nr. 665640) in a 12-well format (growth area: 1.12 cm^2^) that were coated with 0.12 mg/mL rat tail collagen I (Sigma-Aldrich, Vienna, Austria, Cat. Nr. C3867) at 37 °C for 1 h. For this, different cell numbers were used for the four cell lines. RPMI2650: 1 × 10^6^ cells/Thincert®, A549: 5 × 10^5^ cells/thincert, TR146: 1 × 10^5^ cells/thincert, Calu-3: 5 × 10^5^ cells/Thincert®. The medium supplemented with 1 µM hydrocortisone (Sigma-Aldrich, Vienna, Austria, Cat. Nr. H0888) was added to the cells 24 h post-seeding. In the basal compartment, 5.5 mL medium was added to the apical compartment, and 0.5 mL medium was applied. On day 8 ALI was introduced, by removing the apical medium and reducing the basal medium to 4 mL. The medium was changed every 2–3 days, where also TEER was measured, using the EVOM3 (world precision instruments, wpi) combined with chopstick electrodes (STX3 and STX4, world precision instruments, Friedberg, Germany). Experiments were performed as technical replicates (control LLI: *n* = 3; 1 µM hydrocortisone/HC LLI: *n* = 3; control ALI: *n* = 3; 1µM HC ALI: *n* = 3; blank medium *n* = 1). After 8 days of LLI cultivation followed by 14 days of ALI cultivation (total of 22 days) cells were fixed on Thincerts^®^ for staining.

### 4.3. Evaluation of Tight Junction Formation via Immunocytochemistry

After fixation with 4% paraformaldehyde (Sigma-Aldrich, Vienna, Austria, Cat. Nr. P6148) and permeabilized with 0.2% Triton-X 100 (Sigma-Aldrich, Cat. Nr. X100) for 15 min. A 5% BSA (Sigma-Aldrich, Vienna, Austria, Cat. Nr. A2153) was used as a blocking solution. Cells were then stained with 1 µg/mL Hoechst (Invitrogen, Vienna, Austria, Cat. Nr. H1398), 1 unit/mL Phalloidin (Invitrogen, Vienna, Austria, Cat. Nr. 21834), 5 µg/mL mouse-anti-ZO-1 monoclonal antibody (Invitrogen, Vienna, Austria, Cat. Nr. 33-9100) and a 10 µg/mL goat anti-mouse IgG Alexa Fluor™ 488 labeled secondary antibody (Invitrogen, Vienna, Austria, Cat. Nr. A32723) to image cell-cell contacts and tight junction-associated proteins. All dyes and antibodies were diluted in PBS (Sigma-Aldrich, Vienna, Austria, Cat. Nr. D8537) containing 0.5% BSA. Cells were washed three times for 5 min with PBS after blocking and in between staining steps.

### 4.4. Toxicological Evaluation of Zinc Oxide and Titanium Dioxide Nanoparticles

Cells were seeded at 100% and 50% confluency on rat tail collagen I coated 96-well plates (Greiner Bio-One, Vienna, Austria, Cat. Nr. 655101; growth area: 34 mm^2^). RPMI2650: 1.8 × 10^6^ and 9 × 10^4^ cells/well, A549: 1.2 × 10^5^ and 6 × 10^4^ cells/well, TR146: 3 × 10^4^ and 1.5 × 10^4^ cells/well, Calu-3: 6 × 10^4^ and 3 × 10^4^ cells/well. The selection of nanoparticles used for the current study included potentially cytotoxic ZnO (Joint Research Centre, Nanomaterial Repository, Brussels, Belgium, ID: NM62101a, 70–90 nm, uncoated), and pro-inflammatory TiO_2_ (Joint Research Centre, ID: NM01001a, 5–6 nm, Anatase), which were applied in different concentrations (0.125–2 mM) to the four cell lines. Untreated cells were used as a negative control. Presto Blue assay (Thermo Fisher Scientific, Vienna Austria, Cat. Nr. A13261) was used to measure cell viability. Medium in wells was removed and replaced with staining reagent, diluted 1:10 in medium, and incubated for 3 h, according to the manufacturers protocol. Control samples included untreated cells (neg. ctrl.), acellular controls (medium blank), as well as 0.1% TritonX-100 (pos. ctrl. for cytotoxic events). Readout was performed via fluorescence measurements (Excitation: 560 nm; Emission: 590 nm) using an EnSpire 2300 plate reader by PerkinElmer (Vienna, Austria). The same protocol was then applied for toxicology testing on the barrier models after 14 days of ALI on Thincerts^®^.

## 5. Conclusions

In the current study, we demonstrated how culture modalities impact nanotoxicological read-outs. As the next step, we will implement the initial findings of the current study to further optimize the culture conditions for a microfluidic human mucosa-on-a-chip system for nanotoxicological studies not only automating several steps (e.g., medium supply, sensor integration, nanomaterial exposure, etc.). Further optimization of a common ALI cultivation protocol going deeper into the optimization of media supplements and growth factors, as well as the timepoint of ALI initiation as well as a more dynamic culture condition (i.e., perfusion culture), will be investigated to improve the maturation of individual barrier properties on a common platform. This critical step will be paramount for the simultaneous integration of multiple mucosa models that are connected in an anatomically correct network of tissues to better resemble human nanomaterial uptake in vivo. The combination of reliable mature barrier models [79] with fluid perfusion [80,81], as well as airborne particle uptake [47,48] within an air–liquid interface (i.e., using a nebulizer) will further approximate the models to simulate both chronic and acute nanomaterial exposure scenarios relevant for the human health [82]. We believe that further levels of investigation should elaborate more on the underlying molecular mechanisms of nanotoxicology involving DNA/RNA integrity [24], mitochondrial health [25] as well as apoptotic cell signaling [22,23,26,27,28].

## Figures and Tables

**Table 1:** Summary of fundamental characteristics of the four mucosa cell lines including cell length, cell density of a confluent monolayer as well as mean doubling time.

Cell Line	Cell Length [µm, mean ± sdev]	Confluency [cells/cm^2^]	Doubling Time [h]
RPMI2650	19.3 ± 2.3	6 × 10^5^	41.2 ± 9.3
A549	47.4 ± 5.2	4 × 10^5^	34.6 ± 9.0
TR146	222.3 ± 25.9	1 × 10^5^	63.8 ± 17.7
Calu-3	79.1 ± 16.6	1.8 × 10^5^	137.3 ± 20.4

## Data Availability

Data is available under reasonable email request.

## Supplementary Materials

The supporting information can be downloaded at: https://www.mdpi.com/article/10.3390/ijms24065634/s1.
