# Supplementary material for: The Cultivation Modality and Barrier Maturity Modulate the Toxicity of Industrial Zinc Oxide and Titanium Dioxide Nanoparticles on Nasal, Buccal, Bronchial, and Alveolar Mucosa Cell-Derived Barrier Models
Source: Int J Mol Sci. 2023 Mar 15;24(6):5634. doi: 10.3390/ijms24065634 (PMC10056597; doi:10.3390/ijms24065634)
Supplement: Supplementary file 1 [file ijms-24-05634-s001.zip › ijms-2206944-supplementary.pdf]

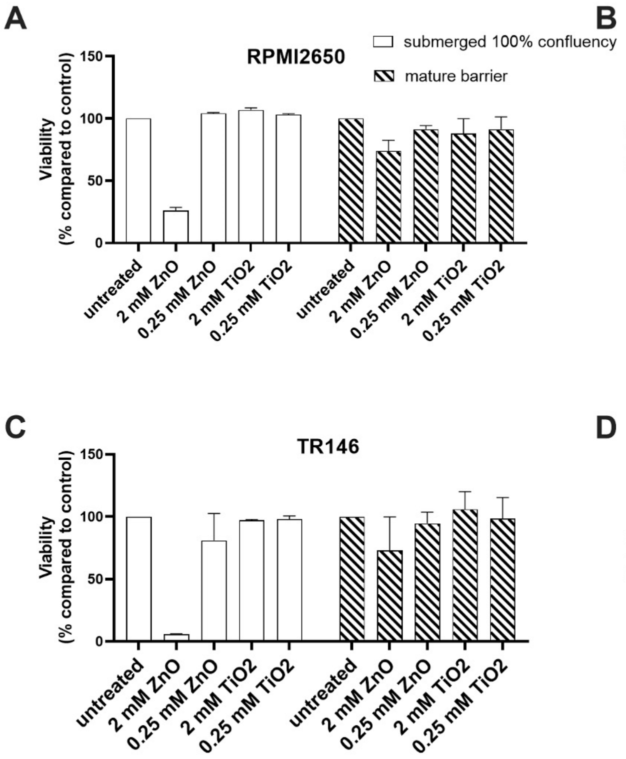

Figure S1: Comparison of conventional 2D monolayer cultures (100% confluent submerged) with mature barrier models grown 22 days under ALI cultivation protocols. (**A**) RPMI2650; (**B**) A549; (**C**) TR146; (**D**) Calu-3.
